# Bone Mass and the CAG and GGN Androgen Receptor Polymorphisms in Young Men

**DOI:** 10.1371/journal.pone.0011529

**Published:** 2010-07-12

**Authors:** Amelia Guadalupe-Grau, Francisco Germán Rodríguez-González, Jesús Gustavo Ponce-González, Cecilia Dorado, Hugo Olmedillas, Teresa Fuentes, Jorge Pérez-Gómez, Joaquín Sanchís-Moysi, Bonifacio Nicolás Díaz-Chico, José A. L. Calbet

**Affiliations:** 1 Department of Physical Education, University of Las Palmas de Gran Canaria, Las Palmas de Gran Canaria, Canary Islands, Spain; 2 Molecular Endocrinology Group, Department of Biochemistry and Physiology, Faculty of Health Sciences, University of Las Palmas of Gran Canaria, Las Palmas de Gran Canaria, Canary Islands, Spain; 3 Canary Islands Cancer Research Institute (ICIC), Las Palmas de Gran Canaria, Canary Islands, Spain; Universidad Europea de Madrid, Spain

## Abstract

**Background:**

To determine whether androgen receptor (AR) CAG (polyglutamine) and GGN (polyglycine) polymorphisms influence bone mineral density (BMD), osteocalcin and free serum testosterone concentration in young men.

**Methodology/Principal Findings:**

Whole body, lumbar spine and femoral bone mineral content (BMC) and BMD, Dual X-ray Absorptiometry (DXA), AR repeat polymorphisms (PCR), osteocalcin and free testosterone (ELISA) were determined in 282 healthy men (28.6±7.6 years). Individuals were grouped as CAG short (CAG_S_) if harboring repeat lengths of ≤21 or CAG long (CAG_L_) if CAG >21, and GGN was considered short (GGN_S_) or long (GGN_L_) if GGN ≤23 or >23. There was an inverse association between logarithm of CAG and GGN length and Ward's Triangle BMC (r = −0.15 and −0.15, P<0.05, age and height adjusted). No associations between CAG or GGN repeat length and regional BMC or BMD were observed after adjusting for age. Whole body and regional BMC and BMD values were similar in men harboring CAG_S_, CAG_L_, GGN_S_ or GGN_L_ AR repeat polymorphisms. Men harboring the combination CAG_L_+GGN_L_ had 6.3 and 4.4% higher lumbar spine BMC and BMD than men with the haplotype CAG_S_+GGN_S_ (both P<0.05). Femoral neck BMD was 4.8% higher in the CAG_S_+GGN_S_ compared with the CAG_L_+GGN_S_ men (P<0.05). CAG_S_, CAG_L_, GGN_S_, GGN_L_ men had similar osteocalcin concentration as well as the four CAG-GGN haplotypes studied.

**Conclusion:**

AR polymorphisms have an influence on BMC and BMD in healthy adult humans, which cannot be explained through effects in osteoblastic activity.

## Introduction

Androgen receptor (AR) are expressed in osteoblasts, osteoclasts, and osteocytes [1], [2]. Hypogonadism in men is associated with increased bone turnover and bone loss [3], and it is reverted by testosterone treatment [4]. However, conflicting results have been reported regarding the association between testosterone and bone mineral density (BMD) in older men [5]–[7]. In men over the age of 65 years with testosterone levels within the normal range, lower concentrations of testosterone were not associated with low bone density [5].

Since some polymorphic variations of the AR confer increased androgen sensitivity [8], [9], differences in the strength of the association between circulating testosterone and bone mass could be explained by differences in the polymorphic variation of the ARs [7].

The AR has a variable NH_2_-terminal domain that contains two functionally polymorphic microsatellites, a polyglutamine tract encoded by CAG repeats and a polyglycine tract (GGN) encoded by (GGT)_3_GGG(GGT)_2_(GGC)_n_ repeats [8]. Long CAG repeats are associated with reduced AR transactivation activity and weaker transcriptional potential [8], while androgen responsiveness is increased in cell cultures with short GGN repeats [9]. However, previous studies on the influence of the CAG repeat polymorphism on BMD have yield conflicting results [7], [10]–[12], maybe due to lack of consideration of a potential interaction between the CAG and GNN repeat polymorphisms.

Thus we hypothesized that the length of CAG and GNN polymorphism will be inversely associated to BMD in men and that CAG and GGN will interact, meaning that men harbouring short alleles of both CAG and GGN repeats will have greater BMD.

The main purpose of this study was to determine whether androgen receptor CAG and GGN polymorphisms influence BMD, bone mineral content (BMC), free serum testosterone concentration and serum osteocalcin concentration, as a marker of osteoblastic function, in young men.

## Methods

### Subjects

Two-hundred eighty-two men (age: 28.6±7.6 years; height: 176.8±5.5 cm; body mass: 78.1±10.3 kg; mean ± SD) participated in the study (Table 1). They were recruited between university students, sports clubs and local police officers in Gran Canaria (Spain). The health status of each participant was established by a medical history and physical examination. Subjects under medication or having any chronic disease or hypertension were excluded.

**Table 1:** Body composition, anthropometrics and physical activity in men with CAG_S_ and CAG_L_ androgen receptor polymorphisms (mean ± standard deviation).

	CAG_S_			n	CAG_L_			n
Age	28.3	±	7.6	151	29.5	±	7.6	131
Height (years)	176.5	±	5.3	151	177.1	±	5.8	131
Body mass (kg)	77.5	±	9.7	151	78.8	±	10.9	131
Percentage of body fat (%)	19.3	±	6.7	151	19.3	±	8.0	131
Sports history (years)	7.9	±	6.3	145	8.1	±	5.3	128
**Hormones**								
Free Testosterone (pg/mL)	21.1	±	11.3	140	21.1	±	9.6	121
Osteocalcin (ng/mL)	21.2	±	7.3	145	21.4	±	9.0	120
**Body composition**								
Whole body BMC (g)	2985.4	±	321.3	151	2997.9	±	362.0	131
Whole body BMD (g/cm^2^)	1.2	±	0.1	151	1.2	±	0.1	131
Upper extremities BMC (g)	386.8	±	56.4	151	395.0	±	55.69	131
Upper extremities BMD (g/cm^2^)	0.8	±	0.1	151	0.8	±	0.0	131
Lower extremities BMC (g)	1266.1	±	149.8	151	1247.2	±	158.5	131
Lower extremities BMD (g/cm^2^)	1.5	±	0.1	151	1.5	±	0.1	131
Femoral neck BMC (g)	6.1	±	0.9	151	5.9	±	1.0	131
Femoral neck BMD (g/cm^2^)	1.0	±	0.1	151	1.0	±	0.1	131
Ward's triangle BMC (g)	1.0	±	0.2	151	0.9	±	0.2	131
Ward's triangle BMD (g/cm^2^)	0.9	±	0.2	151	0.8	±	0.2	131

BMC: bone mineral content, BMD: bone mineral density. Individuals were considered as CAG short (CAG_S_) if harboring repeat lengths ≤21 and CAG long (CAG_L_) if harboring repeat lengths of >21.

### Ethics Statement

The study was performed in accordance with the Helsinki Declaration of 1975, last modified in 2000, as regards the conduct of clinical research, being approved by the Ethical Committee of the University of Las Palmas de Gran Canaria. All volunteers provided their written consent before participation in the study.

### Blood sampling and body composition analysis

The testing day started with a 20 ml blood sample collection from an antecubital vein in the supine position, between 7.30 and 8.30 a.m. Afterwards, whole body composition was assessed by dual-energy X-ray absorptiometry (DXA) (QDR-1500, Hologic Corp., Software version 7.10, Waltham, MA) as reported elsewhere [13], [14].

### Serum free testosterone and osteocalcin determinations

Serum free testosterone and osteocalcin were determined by Enzyme-Linked Immunosorbent Assay (ELISA) (ELx800 Universal Microplate Reader, Bioteck Instruments Inc, Vermont, USA), using reagent kits from IBL (Hamburg, Germany) for free testosterone and from Nordic Bioscience Diagnostics (Herlev, Denmark) for osteocalcin. Low-end sensitivity was 0.17 pg/mL and 0.5 ng/mL for the free testosterone and osteocalcin, respectively. Intra- and interassay coefficients of variation were 6.1 and 7.8% for free testosterone, 6.7 and 6.7% for osteocalcin [15], [16].

### CAG and GGN repeat polymorphisms

DNA was extracted from blood samples (200 µl) using High Pure PCR Template Preparation Kits (Roche Applied Science). To determine the length of the CAG and GGN repeats the corresponding regions located on the exon 1 of the AR gene (Genbank accession no. M27423) were amplified using two pairs of primers whose sequence has been previously reported [17]. One primer from each pair was marked with fluorescent dye (FAM or VIC). Amplification was performed in a 25 µl reaction volume, containing 50 ng of genomic DNA, 200 µM of each deoxynucleotide triphosphate, 1x Fast Start Taq DNA polymerase Buffer (Roche Applied Science, Mannheim, Germany), 1x GC-rich solution buffer (Roche Applied Science) and 1U of Fast Start Taq DNA polymerase (Roche Applied Science). The concentration of each pair of primers was 1.2 and 1.5 µM for the amplification of the CAG and GGN repeats, respectively. PCR conditions were: 30 cycles of 95°C for 45 sec, 56°C for 30 sec and 72°C for 30 sec for CAG amplification; 30 cycles of 95°C for 1 min, 55°C for 2 min and 72°C for 2 min for GGN amplification. Each PCR was initiated with a denaturation step at 95°C for 5 min and terminated with an extension step at 72°C for 5 min. The PCR product was diluted 1:100 in distilled water and 1 µl of the dilution was mixed with 10 µl of formamide and 0.3 µl of GeneScan 500 LIZ Size Standard (Applied Biosystems, Warrington, UK), denatured at 98°C for 5 min and cooled on ice. Fragment separation was performed by automated capillary electrophoresis, using an ABI Prism 3100 Genetic Analyzer (Applied Biosystems) and the length was determined with Gene Scan Analysis Software (version 3.7) (Applied Biosystems).

### Statistics

All variables were checked for normal distribution by the Kolmogorov-Smirnov test. Due to non-normal distribution, CAG and GGN repeat length, osteocalcin and free testosterone were logarithmically transformed. The influence of CAG and GGN repeat lengths on bone mineral content and areal density was determined taking CAG and GGN repeat lengths as either continuous variables or as dichotomous variables with allele cutoff thresholds. Individuals were grouped as CAG short (CAG_S_) if harboring repeat lengths of ≤21 and CAG long (CAG_L_) if harboring repeat lengths of >21. Subjects were ascribed to the GGN short (GGN_S_) group if harboring repeat lengths of ≤23, otherwise they were included in the GGN long (GGN_L_) group. The median value which resulted in the most balanced grouping was used as cutoff threshold [14], [17]. In addition, the subjects were also grouped if having any of the following haplotype combinations: CAG_S_+CGN_L_, CAG_L_+CGN_S_, CAG_L_+CGN_L_ and CAG_S_+CGN_S_. Relationships between variables were assessed calculating the Pearson correlation coefficient (r_p_) in normally distributed variables. The Spearman's correlation coefficient (r_s_) was determined to assess potential linkage disequilibrium between CAG and GGN microsatellites, using the native CAG and GGN values, which were not normally distributed.

Differences between variables were assessed using ANOVA with 2 factors (CAG and GGN lengths), each with two levels (short and long repeat number). In addition, we determined differences between haplotypes using ANOVA with four different haplotype combinations: CAG_S_+CGN_L_, CAG_L_+CGN_S_, CAG_L_+CGN_L_ and CAG_S_+CGN_S_. ANCOVA with age and height as covariates was also used to account for potential confounding of these two variable on bone and hormonal variables. Stepwise multiple regression analysis was used to determine which variables predict femoral neck BMD.

## Results

Subject's body composition and anthropometrics is reported in Table 1. Androgen receptor CAG and GGN repeat lengths were not correlated (Spearman's correlation = −0.05, P = 0.43) (Spearman's correlation = −0.03, P = 0.79), indicating lack of linkage disequilibrium between CAG and GGN microsatellites.

### CAG repeat polymorphism

There was an inverse association between the logarithm of the CAG repeat length and femoral neck BMC, Ward's Triangle BMC and Ward's Triangle BMD (r_p_ = −0.12, −0.14 and −0.12, P<0.05). However, after adjusting for age and height, only the association between the logarithm of the CAG repeat length and Ward's Triangle BMC remained significant (r = −0.15, P<0.05). In the other skeletal regions studied, men with CAG_S_ had BMC and BMD values similar to CAG_L_ men (Table 1).

### GGN repeat polymorphism

No associations between GGN length and regional BMC or BMD were observed. However, after adjusting for age and height there was and inverse association between the logarithm of GGN length and Ward's Triangle BMC (r = −0.15, P<0.05). Regional BMC and BMD were similar between the GGN_S_ and GGN_L_ groups (Table 2).

**Table 2:** Body composition, anthropometrics and physical activity in men with GGN_S_ and GGN_L_ androgen receptor polymorphisms (mean ± standard deviation).

	GGN_S_			n	GGN_L_			n
Age	28.7	±	7.1	170	29.1	±	8.4	112
Height (cm)	176.6	±	5.5	170	177.1	±	5.6	112
Body mass (kg)	77.5	±	9.8	170	79.1	±	10.9	112
Percentage of body fat (%)	19.0	±	6.9	170	19.7	±	7.9	112
Sports history (years)	7.8	±	5.9	165	8.3	±	5.8	108
**Hormones**								
Free Testosterone (pg/mL)	22.1	±	11.2	158	19.6	±	9.3	103
Osteocalcin (ng/mL)	21.4	±	8.4	160	21.3	±	7.7	105
**Body composition**								
Whole body BMC (g)	2969	±	27	170	3024	±	31	112
Whole body BMD (g/cm^2^)	1.24	±	0.01	170	1.25	±	0.01	112
Upper extremities BMC (g)	387	±	56	170	395	±	56	112
Upper extremities BMD (g/cm^2^)	1248	±	158	170	1272	±	147	112
Lower extremities BMC (g)	0.83	±	0.05	170	0.84	±	0.05	112
Lower extremities BMD (g/cm^2^)	1.47	±	0.12	170	1.47	±	0.10	112
Femoral neck BMC (g)	6.0	±	0.1	170	6.1	±	0.1	112
Femoral neck BMD (g/cm^2^)	1.04	±	0.01	170	1.02	±	0.01	112
Ward's triangle BMC (g)	1.0	±	0.0	170	0.9	±	0.0	112
Ward's triangle BMD (g/cm^2^)	0.89	±	0.01	170	0.85	±	0.01	112

BMC: bone mineral content, BMD: bone mineral density. Subjects were considered as GGN short (GGN_S_) if harboring repeat lengths ≤23 and GGN long (GGN_L_) if harboring repeat lengths >23.

### CAG and GGN repeat polymorphisms haplotypes

**Figure 1:**
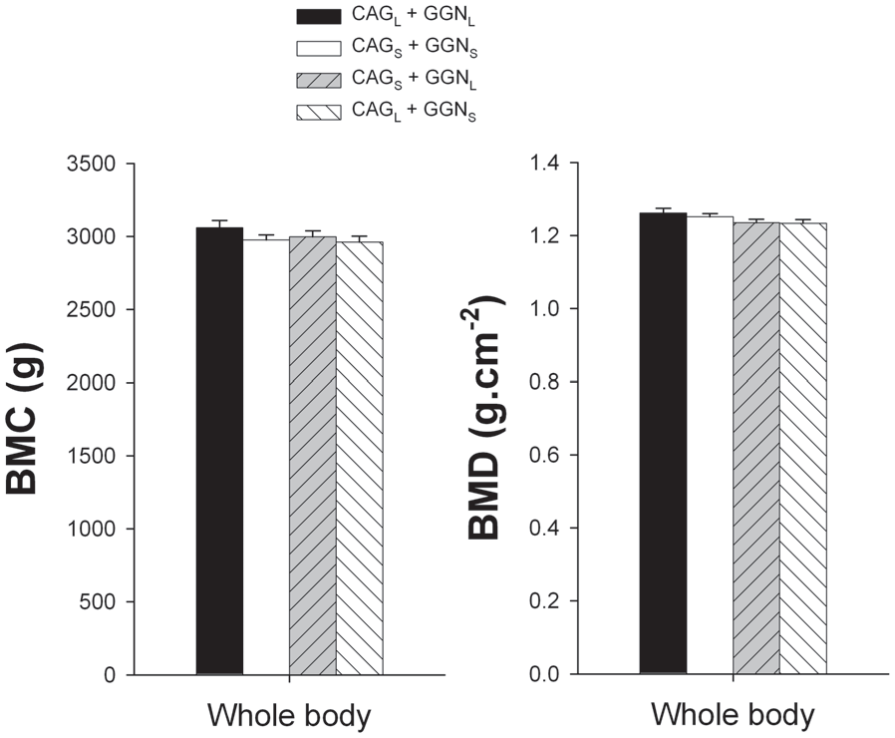
Whole body bone mineral content (BMC) and bone mineral areal density (BMD). Subjects were grouped as CAG short (CAG_S_) if harboring repeat lengths of ≤21 and CAG long (CAG_L_) if harboring repeat lengths of >21. The cutoff point for GGN short (GGN_S_) was GGN repeat polymorphism ≤23, otherwise subjects were included in the GGN long (GGN_L_) group. Four haplotypes combinations were defined as: CAG_L_ + GGN_L_, CAG_S_ + GGN_S_, CAG_S_ + GGN_L_, and CAG_L_ + GGN_S_.

**Figure 2:**
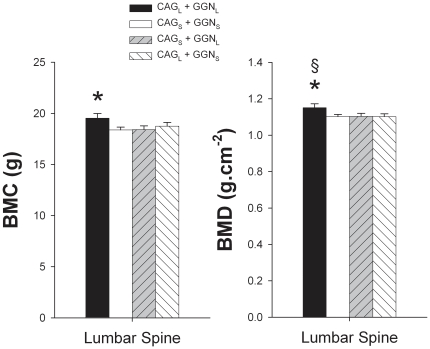
Mean lumbar spine (from L_2_, L_3_ and L_4_) bone mineral content (BMC) and bone mineral areal density (BMD). Subjects were grouped as CAG short (CAG_S_) if harboring repeat lengths of ≤21 and CAG long (CAG_L_) if harboring repeat lengths of >21. The cutoff point for GGN short (GGN_S_) was GGN repeat polymorphism ≤23, otherwise subjects were included in the GGN long (GGN_L_) group. Four haplotypes combinations were defined as: CAG_L_ + GGN_L_, CAG_S_ + GGN_S_, CAG_S_ + GGN_L_, and CAG_L_ + GGN_S_. * P<0.05 compared to CAG_S_ + GGN_S_; § P<0.05 compared to CAG_L_ + GGN_S_

**Figure 3:**
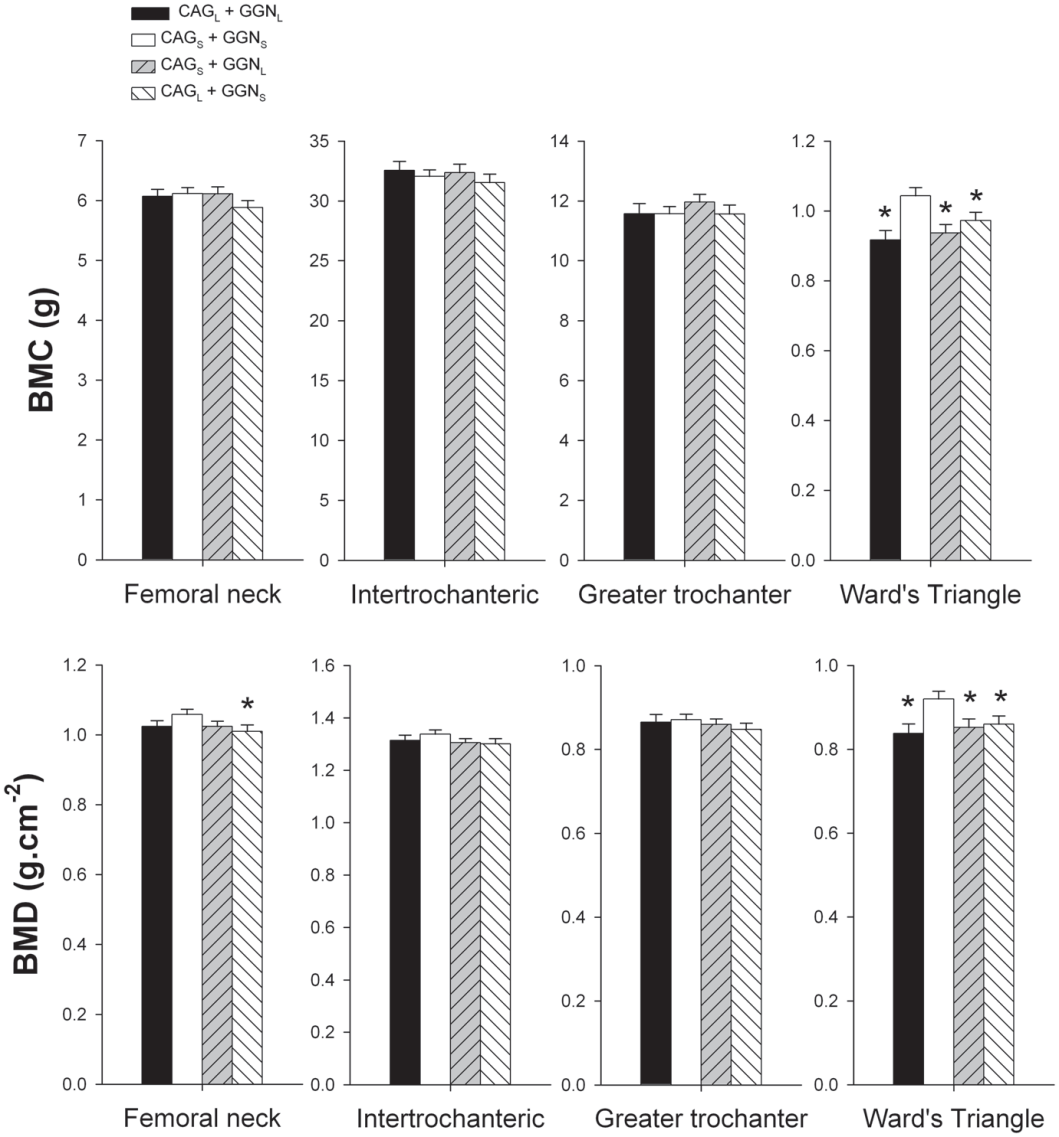
Hip bone mineral content (BMC) and bone mineral areal density (BMD). Subjects were grouped as CAG short (CAG_s_) if harboring repeat lengths of ≤21 and CAG long (CAG_L_) if harboring repeat lengths of >21. The cutoff point for GGN short (GGN_s_) was GGN repeat polymorphism ≤23, otherwise subjects were included in the GGN long (GGN_L_) group. Four haplotypes combinations were defined as: CAG_L_ + GGN_L_, CAG_S_ + GGN_S_, CAG_S_ + GGN_L_, and CAG_L_ + GGN_S_. * P<0.05 compared to CAG_S_ + GGN_S_; § P<0.05 compared to CAG_L_ + GGN_S_.

**Figure 4:**
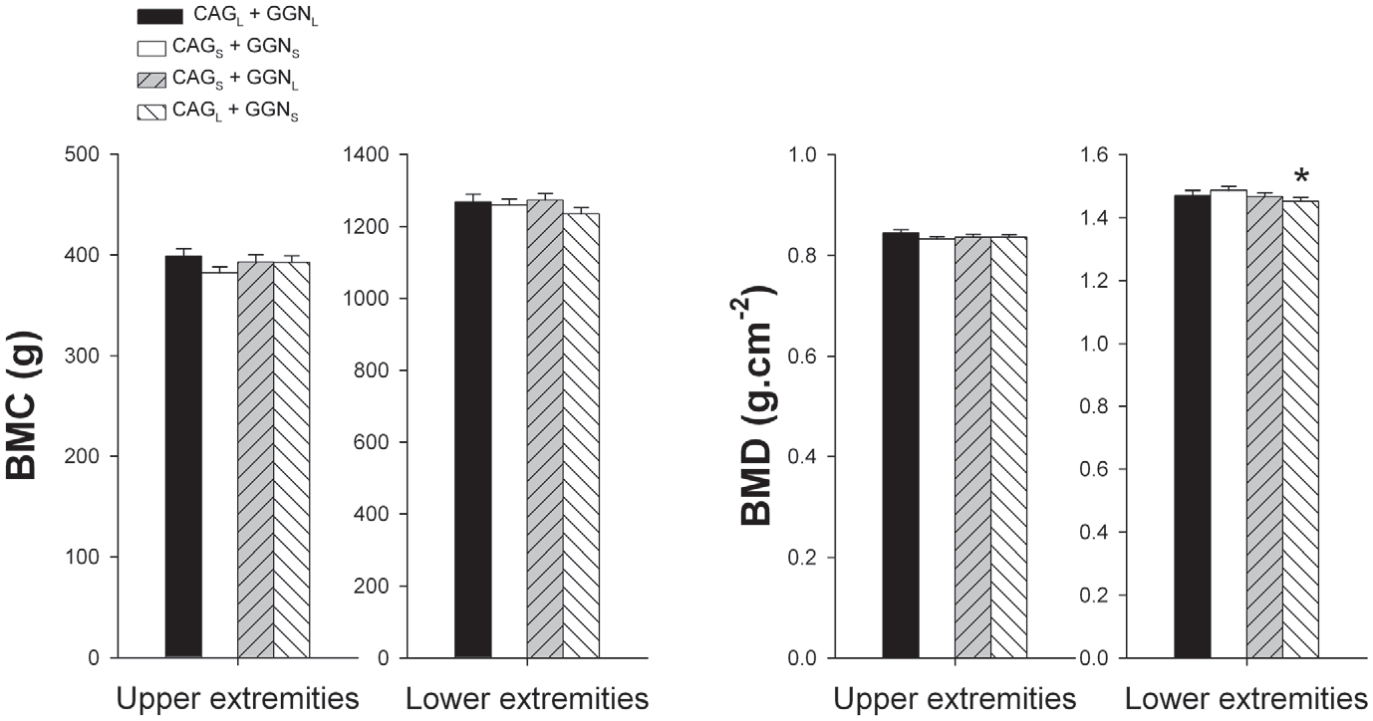
Bone mineral content (BMC) and areal bone mineral density (BMD) of the upper and lower extremities. Subjects were grouped as CAG short (CAG_S_) if harboring repeat lengths of ≤21 and CAG long (CAG_L_) if harboring repeat lengths of >21. The cutoff point for GGN short (GGN_S_) was GGN repeat polymorphism ≤23, otherwise subjects were included in the GGN long (GGN_L_) group. Four haplotypes combinations were defined as: CAG_L_ + GGN_L_, CAG_S_ + GGN_S_, CAG_S_ + GGN_L_, and CAG_L_ + GGN_S_. * P<0.05 compared to CAG_S_ + GGN_S_; $ P<0.05 compared to CAG_L_ + GGN_L_.

Whole body BMC and BMD were similar in the four CAG-GGN haplotypes studied (Fig. 1). Men harboring the combination CAG_L_+GGN_L_ had 6.3% higher lumbar spine BMC than men with the haplotype CAG_S_+GGN_S_ (P<0.05) (Fig. 2). Likewise, men with the haplotype CAG_L_+GGN_L_ had 4.4 and 4.3% higher lumbar spine BMD than men harboring the combinations CAG_S_+GGN_S_ and CAG_L_+GGN_S_, respectively (both P<0.05) (Fig. 2). Men with the combination CAG_S_ + GGN_S_ had 13.8, 11.7 and 7.2% higher Ward's Triangle BMC than those with the combinations CAG_L_+GGN_L_, CAG_S_+GGN_L_, and CAG_L_+GGN_S_, respectively (all P<0.05) (Fig. 3). Ward's Triangle BMD was 9.8, 7.9 and 6.9% higher in men with CAG_S_+GGN_S_ compared to CAG_L_+GGN_L_, CAG_S_+GGN_L_, and CAG_L_+GGN_S_, respectively (all P<0.05) (Fig. 3). Femoral neck BMD was 4.8% higher in the CAG_S_+GGN_S_ compared with the CAG_L_+GGN_S_ men (P<0.05) (Fig. 3). The mean BMD of the lower extremities was 2.4% higher in the CAG_S_+GGN_S_ compared with the CAG_L_+GGN_S_ men (P<0.05) (Fig. 4). All these between-haplotypes differences in regional BMD and BMC remained statistically significant after accounting for height and age as covariates.

### Free testosterone bone mass and androgen receptor polymorphism

There was no relationship between either CAG or GGN repeats length and free testosterone, even after adjusting for age. Basal serum free testosterone concentrations were similar in CAG_S_ and CAG_L_ (21.1±11.3 and 21.1±9.6 pmol/L, respectively P = 0.43). Although free testosterone concentration was 13% higher in GGN_S_ compared to GGN_L_ this difference did not reach statistical significance (22.1±11.2 and 19.6±9.3 pmol/L, respectively, P = 0.07). There were significant associations between the logarithm of free testosterone concentration and BMC at the whole body, femoral neck, Ward's Triangle, and lower extremities (r_p_ = 0.15, 0.21, 0.18, and 0.12, respectively, P<0.05). The logarithm of free testosterone concentration was also associated to BMD at the femoral neck, Intertrochanteric region, Greater trochanter, Ward's Triangle, and lower extremities (r_p_ = 0.21, 0.13, 0.20, 0.22, and 0.17, respectively, all P<0.05). However, after adjusting for age there was no association between the logarithm of free testosterone concentration and regional or whole body BMC and BMD.

Free testosterone concentrations were similar across the four haplotypes (i.e., 20.1±9.1, 22.4±12.4, 19.3±9.5, and 21.7±10.0, for CAG_L_+CGN_L_, CAG_s_+CGN_s_, CAG_s_+CGN_L_, and CAG_L_+CGN_s_, respectively).

### Androgen receptor polymorphisms and osteoblastic function

There was no relationship between either CAG or GGN repeats length and osteocalcin. As shown in Table 1, serum osteocalcin concentrations were similar in CAG_S_ and CAG_L_ (Table 1) as well as in GGN_S_ and GGN_L_ subjects (Table 2). Osteocalcin concentrations were also similar across the four haplotypes (data not shown). However, there were significant correlations between osteocalcin and: free testosterone, BMC at femoral neck, greater trochanter, intertrochanteric area, Ward's Triangle, BMD at whole body, femoral neck, greater trochanter, intertrochanteric area, (r_p_ = 0.23, 0.27, 0.19, 0.28, 0.13, 0.31, 0.32 and 0.23, respectively, P<0.05). All these correlations were not significant after adjusting for age, due to the negative correlation between the logarithm of osteocalcin and age (r_p_ = −0.57, P<0.01).

## Discussion

This study indicates that androgen receptor polymorphisms have an influence on bone mass and density in healthy adult men. These associations showed regional specificity, implying that the relationship between androgen receptor polymorphisms and bone mass is modulated, and in some instances overridden, by other endocrine variables and local factors. We have found an inverse association between CAG and GGN repeat length and Ward's Triangle BMC. However, the most interesting aspect of this study is that it shows that there is a regional specificity in the relationship between the CAG and GGN polymorphisms and bone mass. In contrast with our hypothesis, men with CAG_L_+GGN_L_ haplotypes, which potentially have lower androgen responsiveness, have greater BMC and BMD at the lumbar spine than men harboring CAG_S_+GGN_S_ haplotypes. However, femoral neck BMD was higher in men with CAG_S_+GGN_S_ compared with CAG_L_+GGN_S_ haplotypes.

### Bone mass and androgen receptor CAG and GNN repeat polymorphism

When CAG and GGN polymorphism were considered separately, after adjusting for age and height, no association between CAG and GGN length and bone mineral density was observed in the present investigation. The influence of GGN repeat AR polymorphism on bone mass and density has not been studied previously and, hence, we can not compare our results with other studies. Conflicting results have been reported regarding the influence of CAG repeat length on BMD. In agreement with our results, lack of association between CAG repeat length and BMD has been reported in middle aged [12] and aged men [18]. However, a positive association between BMD of the lumbar spine and femoral neck with length of the CAG repeat polymorphism has also been reported in men over the age of 40 years [7], [11].

Some of these discrepancies just reflect a polygenic influence on bone mass and the impact of a number of factors, such as, age, gender, nutrition, race, life style, mechanical loading, skeletal region under consideration among others, which are known to influence bone metabolism [5], [19]–[22]. This is further underlined by the fact that even studies in the same population, for example Scandinavians, but carried out in different countries have yield different results [7], [10]–[12]. In the studies in which an association have been found between CAG repeat length polymorphism and BMD, its strength has consistently been rather low, i.e., CAG repeat length polymorphism can only explain a rather small part of the variance in BMD, and only in some specific regions. Thus, other factors influencing BMD prevail on CAG repeat polymorphism in many instances.

### Free testosterone and androgen receptor polymorphism

Previous studies have reported lack of association between CAG repeat number and free testosterone in middle-aged [7], [23] and aged men [18]. The present investigation shows that also in young adult men there is no association between CAG or GGN length and free testosterone levels. As expected, there was a positive association between free testosterone levels and bone mass and density in men [6]. However, this association was not statistically significant after adjusting for age.

A relationship between serum testosterone levels and CAG repeat length and femoral BMD has been recently reported in healthy Swedish men [7]. These authors observed that men having the combination of low free testosterone and shorter CAG repeat length had the lower femoral neck BMD [7]. After adjusting for age we could not confirm this association between free testosterone, CAG repeat length and femoral neck BMD. A part from the different age of the populations, racial and environmental differences could also explain the discrepancies.

### Osteocalcin and androgen receptor polymorphisms

Although androgen receptors repeat polymorphisms have an influence on BMC and BMD in healthy adult humans, this could not be explained by differences in osteoblastic activity. In concordance with our results, Valimaki et al [24], measured markers of bone turnover in a cohort of males with similar ages as ours (18–21yrs), and did not find any difference between individuals carrying either short or long CAG repeat lengths. However, the mean CAG repetition number for this cohort was 28, whereas it was 22 in the present investigation, making comparisons between both studies difficult. Additional studies carried out in elders showed no relationship between CAG repeat length and C-terminal telopeptide of the type I collagen, osteocalcin, BSAP and urinary deoxypirindoline [18], [25]. To our knowledge, there are no data available about the influence of GGN repeats on BMD and bone formation/resorption markers precluding any comparisons of our study with previous investigations.

In summary this study shows that there is an association between CAG (and to lesser extend GGN) androgen receptors polymorphisms and bone mass and bone mineral density, which is modulated depending on the age and osteocalcin and free testosterone serum concentrations. Femoral neck BMD is higher in men with CAG_S_+GGN_S_ haplotypes, whilst men harboring CAG_L_+GGN_L_ haplotypes have the higher lumbar spine BMC and BMD.
